# Breakfast skipping and depressive symptoms in an epidemiological youth sample in Hong Kong: the mediating role of reduced attentional control

**DOI:** 10.3389/fpsyt.2025.1574119

**Published:** 2025-05-22

**Authors:** Stephanie Ming Yin Wong, Olivia Choi, Yi Nam Suen, Christy Lai Ming Hui, Edwin Ho Ming Lee, Sherry Kit Wa Chan, Eric Yu Hai Chen

**Affiliations:** ^1^ Department of Social Work and Social Administration, The University of Hong Kong, Hong Kong, Hong Kong SAR, China; ^2^ Department of Psychiatry, School of Clinical Medicine, LKS Faculty of Medicine, The University of Hong Kong, Hong Kong, China; ^3^ School of Nursing, LKS Faculty of Medicine, The University of Hong Kong, Hong Kong, Hong Kong SAR, China; ^4^ The State Key Laboratory of Brain and Cognitive Sciences, The University of Hong Kong, Hong Kong, Hong Kong SAR, China; ^5^ Centre for Youth Mental Health, Faculty of Medicine, Dentistry and Health Sciences, University of Melbourne, Parkville, VIC, Australia; ^6^ Orygen Youth Health, Parkville, VIC, Australia

**Keywords:** breakfast skipping, depressive symptoms, anxiety symptoms, attentional impulsivity, attentional control, youth mental health

## Abstract

**Introduction:**

Breakfast skipping is common among young people, although previous work has suggested its negative influences on cognitive and executive functions and mental health outcomes. Whether reduced impulse control, particularly in the cognitive domain, would be a mechanism that links breakfast skipping to elevated psychiatric symptoms remains to be investigated.

**Methods:**

We used data from 3154 young people (aged 15–25 years) in the Hong Kong Youth Epidemiological Study of Mental Health 2019–2022. Participants were asked about their general breakfast consumption habits, impulsivity (overall and its subdomains, using the Barratt Impulsiveness Scale–11), depressive symptoms (Patient Health Questionnaire–9), anxiety symptoms (Generalized Anxiety Disorder Scale–7), and functioning (reduced and lost productivity due to mental health problems, and the observer-rated Social and Occupational Functioning Assessment Scale). Impulsivity and its subdomains were tested for their potential mediating influences between breakfast skipping frequency and symptom outcomes. Sociodemographic variables, psychiatric history, and eating disorder symptoms were adjusted for in all mediation models.

**Results:**

The weighted prevalence of daily breakfast consumption and breakfast skipping (defined as no breakfast consumption at all) was 33% and 14.2%, respectively. More frequent breakfast skipping was associated with higher levels of impulsivity, specifically in terms of attentional control (*r*=0.14) and self-control (*r*=0.13), and depressive symptoms (*r*=0.14), all *p*<0.001. Breakfast skipping frequency also showed significant associations with anxiety symptoms and poorer functioning, although their relationships were weak (*r* range=0.04–0.08). In a parallel mediation model, attentional impulsivity *(B*=0.21, SE=0.03, CI=0.15–0.27), but not self-control impulsivity (*B*=0.01, SE=0.01, CI=-0.02–0.03), significantly mediated the relationship between breakfast skipping and depressive symptoms and explained 34.2% of the total effect. All findings remained unchanged even when excluding those who reported rising at 12 pm or after.

**Conclusion:**

Breakfast skipping is associated with elevated depressive symptoms in young people, with impaired attentional control being an important mechanism in this relationship. Encouraging young people to build regular breakfast habits may be incorporated as part of future lifestyle interventions for mental disorders and be further emphasised in public health policies.

## 1 Introduction

Breakfast constitutes one of the most important meals of the day (1). After an overnight fast, blood glucose concentration tends to be at its lowest upon waking, which could negatively affect daytime cognitive functions if not restored (2, 3). Cascades of biochemical reactions involving not only depleted blood glucose and impaired glucose homeostasis but also their related physiological mechanisms (e.g., insulin, cortisol, serotonin, and glutamate) can result due to breakfast skipping (4, 5). Irregular breakfast consumption has been shown to be linked to elevated risks of cardiovascular diseases (6), as well as mental health problems, particularly depressive symptoms (7–11), in a range of studies. Yet, skipping breakfast has become increasingly common in today’s society, particularly among young people (12–14).

While youth signifies a time of increased opportunities for social network building and the crystallisation of self-concepts (15), the marked neurobiological, social, physical, and psychological changes that take place during this life stage also make young people more vulnerable to the onset of mental disorders (16, 17). Despite the significant burden of disease associated with depression and anxiety in youth (18), mental health help-seeking remains a persistent challenge. In our previous youth epidemiological study in Hong Kong (19), we found that only 16.7% of those with a 12-month major depressive episode were utilising any psychiatric or psychological services, with financial costs and accessibility difficulties reported as two leading causes of non-utilisation. Similar observations of non-help-seeking among youths have been reported in other countries (20). In addition to enhancing youth-friendly and evidence-based services in the community, determining the possible benefits of building healthy habits, such as regular breakfast consumption, in mental health may offer more practical and low-stigma strategies for well-being in the population.

Among existing studies that have explored the effects of breakfast skipping on young people, the majority focused on cognitive performance deficits in school-aged children (4). A related systematic review found that children and adolescents who do not consume breakfast tend to show poorer cognitive performance of the brain, particularly in tasks involving attention, memory, and executive functions (4). The researchers, nevertheless, noted the relative scarcity of studies conducted on adolescent samples, thereby limiting the generalisability of findings to this age group. More recently, a meta-analysis has suggested that breakfast skipping is associated with elevated depressive and anxiety symptoms in adolescents, with no associations with anxiety symptoms observed in other age groups (7). These studies generally suggested that breakfast consumption plays a role in cognitive functions and mental health symptoms, although no study has yet simultaneously investigated the effects of breakfast skipping on both cognitive functions and mental health outcomes in young people, as well as their possible interrelationships.

Notably, an earlier national Japanese survey of high school students reported that those who skipped breakfast showed significantly higher levels of impulsivity (21). It is possible that breakfast skipping could be linked to deficits in inhibitory control processes [which are closely related to glucose imbalances (22)] and, in turn, affect mental well-being. Indeed, problems in the ability to focus attention and control impulses have long been linked to a vast range of mental health problems (23–26), particularly in young people (27). Whether reduced impulse control may serve as a mechanism that links breakfast skipping to mental health symptoms, even when adjusting for factors that might confound breakfast consumption patterns and their associations with mental health outcomes (e.g., pre-existing mental health problem, symptoms of eating restraints, and later rise time), warrants further investigation.

Extending observations in previous work, we conducted this study with three major aims: to determine the patterns of breakfast consumption in a population-representative household-based youth sample in Hong Kong; to investigate the associations between breakfast skipping, impulsivity, as well as depressive and anxiety symptoms and functioning outcomes; and to explore whether specific domains of impulsivity would mediate the effects of breakfast skipping on poorer mental health outcomes. We hypothesised that young people who more frequently skip breakfast would show poorer outcomes and that impulsivity, particularly in the cognitive and attentional domains, would mediate the effects of breakfast skipping on both depressive and anxiety symptoms.

## 2 Materials and methods

### 2.1 Participants and study design

Participants were from the Hong Kong Youth Epidemiological Study of Mental Health (HK-YES) (19, 28), which was, to date, the first territory-wide household-based study of mental health in Hong Kong specifically targeting young people aged 15–24 years in the city. As in previous large-scale epidemiological studies, a stratified multistage cluster sampling design was adopted to improve sample representativeness. Invitation letters were sent to randomly selected addresses obtained from the local government to all young people within the age group at the time of recruitment. All data were collected from May 2019 to June 2022 by trained research staff through face-to-face interviews or online video conferencing during COVID-19, following the same procedures. Details of the HK-YES have been reported (19, 28–30). Data collected regarding patterns of breakfast consumption habits, impulsivity, depressive and anxiety symptoms, functioning, sociodemographic factors, and plausible confounders in the HK-YES were analysed in the present study. Written informed consent was obtained from all participants in the HK-YES, with additional parental or guardian consent obtained from those below the age of 18 years. Ethical approval was granted by the Institutional Review Board of the University of Hong Kong/Hospital Authority Hong Kong West Cluster.

### 2.2 Measures

#### 2.2.1 Breakfast consumption patterns

Breakfast consumption was assessed using the item “Which of the following best describes your general breakfast habit?”. The item was rated on a 4-point scale with the following options: “eat breakfast daily”, “often eat breakfast”, “rarely eat breakfast”, and “never eat breakfast”. A higher score reflects a habit of more frequent breakfast skipping.

#### 2.2.2 Impulsivity

Impulsivity was assessed using the 30-item Barratt Impulsiveness Scale, version 11 (BIS-11) (31), which has been referred to as the gold standard in the measure of general impulse control (32). All items on the BIS-11 were rated on a 4-point Likert scale (from “rarely/never” to “almost always/always”) and were summed to generate a composite score, wherein a higher score reflects higher impulsiveness (range = 0–90). Aside from overall impulsivity, the BIS-11 comprises six first-order components reflecting various subdomains of impulse control, namely attention (e.g., “I concentrate easily” [reversed]) (range = 0–15); cognitive instability (e.g., “I have ‘racing’ thoughts”) (range = 0–9); motor (“I do things without thinking”) (range = 0–21); perseverance (e.g., “I can only think about one thing at a time”) (range = 0–12); cognitive complexity (e.g., “I like to think about complex problems” [reversed]) (range = 0–15); and self-control (e.g., “I plan tasks carefully”) (range = 0–18). The Chinese version of the BIS-11 has been validated in young people (33) and shows good internal consistency in our sample (α = 0.76). 

#### 2.2.3 Depressive and anxiety symptoms

Depressive and anxiety symptoms during the past two weeks were assessed using the Patient Health Questionnaire–9-item (PHQ-9) (34) and the Generalized Anxiety Disorder–7-item (GAD-7) (35), respectively. Items of the scales were developed based on the DSM-IV criteria, which correspond to symptoms of major depressive disorder and generalised anxiety disorder in the DSM-V. All items were rated on a 4-point Likert scale (from “not at all” to “nearly every day”) and were summed to generate composite scores of depressive and anxiety symptom severity, with higher scores reflecting greater symptom severity]) (PHQ-9 range = 0–27; GAD-7 range = 0–21). Both the PHQ-9 and GAD-7 have been validated in Hong Kong and youth populations (36–38).

#### 2.2.4 Functioning

Three indicators of functioning were assessed, including two items which directly asked the participants the number of days during the past 30 days they have experienced reduced and lost productivity due to psychiatric symptoms, respectively (19) (range = 0–30), and the Social and Occupational Functioning Assessment Scale (SOFAS) (39), which is an interviewer-rated measure derived from the DSM (rated from 0 to 100). A higher score reflects more optimal social and occupational functioning on the SOFAS.

#### 2.2.5 Covariates

Several additional variables were taken as covariates in the study. Sociodemographics include sex, age, socioeconomic status [as reflected by any governmental subsidy received (19)], and any history of a psychiatric disorder. Given possible influences of dietary, shape, and weight concerns in breakfast skipping, symptoms of eating disorders were also included as a plausible confounder, which was reflected by the mean score on the Eating Disorder Examination Questionnaire 6.0 (EDE-Q) (40, 41). Participants were also asked about their regular rising time, which was used to adjust for potential influences of breakfast skipping due to late rising in our sensitivity analyses.

### 2.3 Statistical analysis

The prevalence of breakfast consumption patterns of the general youth population in Hong Kong was first established with weighting adjustments applied according to sex and age data from the local 2019 Census. A series of independent t-tests or Chi-square tests (for continuous and categorical variables) were applied to explore differences across the various domains of impulsivity, depressive and anxiety symptoms, and functioning between those who at least consume breakfast sometimes and those who completely skip breakfast. A series of correlation analyses were then conducted to examine the degree of associations between breakfast skipping frequency, impulsivity (overall and subdomains), mental health symptoms, and functioning. Mediation models were applied to examine the potential mediating effects of impulsivity between breakfast skipping and symptom outcomes [only among variables showing a coefficient of ≥0.1 in the correlation analyses (42)], whilst adjusting for sociodemographic characteristics and eating disorder symptoms.

As a sensitivity analysis, we conducted the same set of analyses after excluding participants who reported regularly waking at 12 pm or after (late risers) to ensure the breakfast skipping was not the result of late rising time. All mediation effects were tested with a bootstrapping of 10,000 samples and a confidence interval of 95%. Analyses were conducted using SPSS version 29.0, with mediation analyses conducted using the PROCESS macro. The statistical significance was set at the *p* < 0.05 level for all analyses.

## 3 Results

### 3.1 Breakfast skipping: prevalence and associated factors

**Table 1** presents the sample characteristics, with a detailed summary of the various psychiatric conditions reported by young people provided in **Supplementary Table S1**. With weighting adjustments applied, 33% of the youth population reported having a habit of consuming breakfast every day. 28.1% often consume breakfast, 24.1% rarely consume breakfast, and 14.8% completely skip breakfast.

**Table 1:** Sample characteristics in the whole sample and by breakfast consumption behaviour.

	Whole sample (n = 3154)	Breakfast consumption (daily/intermittent) (n = 2671)	Breakfast skipping (no consumption) (n = 483)	*p*
Sociodemographic variables and covariates				
Female sex, n (%)	1831 (58.1%)	1542 (57.7%)	289 (59.8%)	0.39
Age	19.8 (2.8)	**19.8 (2.8)**	**20.1 (2.6)**	**0.015**
Any psychiatric history, n (%)	273 (8.7%)	222 (8.3%)	51 (10.6%)	0.11
Any government subsidy received, n (%)	296 (9.4%)	246 (9.2%)	50 (10.4%)	0.43
Eating disorder symptoms (EDE-Q)	1.03 (1.00)	**1.00 (0.97)**	**1.17 (1.14)**	**0.003**
Impulsivity symptoms				
Overall impulsivity (BIS-11)	63.55 (8.44)	**63.16 (8.41)**	**65.70 (8.28)**	**<0.001**
Attention	10.96 (2.23)	**10.87 (2.23)**	**11.48 (2.13)**	**<0.001**
Cognitive instability	5.72 (1.59)	**5.67 (1.59)**	**5.98 (1.58)**	**<0.001**
Motor	13.67 (2.86)	**13.58 (2.83)**	**14.14 (3.00)**	**<0.001**
Perseverance	7.19 (1.53)	**7.16 (1.53)**	**7.38 (1.49)**	**0.004**
Self-control	14.06 (3.06)	**13.97 (3.08)**	**14.58 (2.95)**	**<0.001**
Cognitive complexity	11.95 (2.21)	**11.92 (2.19)**	**12.14 (2.34)**	**0.040**
Mental health symptoms				
Depressive symptoms (PHQ-9)	6.50 (5.20)	**6.25 (5.06)**	**7.88 (5.70)**	**<0.001**
Anxiety symptoms (GAD-7)	4.72 (4.50)	**4.60 (4.44)**	**5.38 (4.77)**	**<0.001**
Functioning				
Days of reduced productivity	2.21 (4.74)	**2.08 (4.51)**	**2.94 (5.80)**	**0.002**
Days of lost productivity	0.41 (2.15)	0.39 (2.04)	0.56 (2.66)	0.18
Social and occupational functioning (SOFAS)	82.56 (7.79)	**82.86 (7.68)**	**80.93 (8.22)**	**<0.001**

Values are presented in the form of mean (SD) or n (%). Statistics significant at the level of *p* < 0.05 are in boldface. BIS, Barratt Impulsiveness Scale; EDE-Q, Eating Disorder Examination Questionnaire; GAD-7, Generalized Anxiety Disorder–7-item; PHQ-9, Patient Health Questionnaire–9-item; SOFAS, Social and Occupational Functioning Assessment Scale.

Differences in sociodemographic variables, impulsivity, as well as mental health and functioning outcomes, between those who at least sometimes consume breakfast (rarely to daily) and those who completely skip breakfast are also shown in **Table 1**. Young people who skipped breakfast showed higher levels of overall impulsivity as compared with those who consumed breakfast (mean = 65.70 [SD = 8.28] vs 63.16 [8.41]), *p* < 0.001. The same pattern of findings was observed for each subdomain of impulsivity (**Table 1**). Those who skipped breakfast also showed higher levels of depressive symptoms (mean = 7.88 [SD = 5.70] vs 6.25 [5.06]) and anxiety symptoms (5.38 [4.77] vs 4.60 [4.44]), more days of reduced productivity (2.94 [5.80] vs 2.08 [4.51]), as well as poorer social and occupational functioning (80.93 [8.22] vs 82.86 [7.68]), all *p* < 0.01. They were also slightly older and reported more symptoms of eating disorders. Participant sex, psychiatric history, and socioeconomic status were not different between those who consumed and skipped breakfast, all *p* > 0.05 (**Table 1**).

**Table 2** shows findings from the correlation analyses. Breakfast skipping frequency was associated with higher levels of overall impulsivity (BIS-11, *r* = 0.16), and specifically attentional (*r* = 0.14) and self-control (*r* = 0.13) impulsivity, all *p* < 0.001. The associations between breakfast skipping frequency and other subdomains of impulsivity were significant but very weak, with coefficients below 0.1. Breakfast skipping frequency, overall impulsivity, and both attention and self-control impulsivity were also significantly associated with depressive symptoms (*r* = 0.14; *r* = 0.33; *r* = 0.36; *r* = 0.21), respectively. Moderate degrees of associations were observed between impulsivity and anxiety symptoms and poorer functioning, while very weak associations of breakfast skipping frequency with these outcomes were observed (**Table 2**). Mediation analyses were thus conducted only on depressive symptoms as the outcome.

**Table 2:** Correlations between breakfast skipping frequency, impulsivity, mental health symptoms, and functioning.

	1	2	3	4	5	6	7	8	9	10	11	12	13
Breakfast skipping													
1. Breakfast skipping frequency	–	–	–	–	–	–	–	–	–	–	–	–	–
Impulsivity													
2. Overall impulsivity (BIS-11)	0.16***	–	–	–	–	–	–	–	–	–	–	–	–
3. Attention	0.14***	0.74***	–	–	–	–	–	–	–	–	–	–	–
4. Cognitive instability	0.09***	0.50***	0.36***	–	–	–	–	–	–	–	–	–	–
5. Motor	0.09***	0.62***	0.27***	0.41***	–	–	–	–	–	–	–	–	–
6. Perseverance	0.05**	0.52***	0.28***	0.20***	0.15***	–	–	–	–	–	–	–	–
7. Self-control	0.13***	0.73***	0.54***	0.11***	0.17***	0.30***	–	–	–	–	–	–	–
8. Cognitive complexity	0.08***	0.55***	0.26***	**0.01**	0.15***	0.24***	0.35***	**–**	**–**	**–**	**–**	**–**	**–**
Mental health symptoms													
9. Depressive symptoms (PHQ-9)	0.14***	0.33***	0.36***	0.35***	0.09***	0.20***	0.21***	0.12***	–	–	–	–	–
10. Anxiety symptoms (GAD-7)	0.08***	0.23***	0.27***	0.35***	0.06**	0.14***	0.09***	0.07***	0.75***	–	–	–	–
Functioning													
11. Days of reduced productivity	0.04*	0.12***	0.14***	0.17***	**0.02**	0.09***	0.07***	**0.02**	0.41***	0.37***	**–**	**–**	**–**
12. Days of lost productivity	0.03	0.09***	0.08***	0.15***	0.04*	0.07***	**0.03**	**0.01**	0.27***	0.26***	0.38***	–	–
13. Social and occupational functioning (SOFAS)	-0.08***	-0.16***	-0.15***	-0.16***	**-0.03**	-0.15***	-0.11***	-0.06***	-0.38***	-0.35***	-0.31***	-0.26***	–

Statistics significant at the level of *p* < 0.05 are in boldface. BIS, Barratt Impulsiveness Scale; GAD-7, Generalized Anxiety Disorder–7-item; PHQ-9, Patient Health Questionnaire–9-item; SOFAS, Social and Occupational Functioning Assessment Scale.

*p < 0.05, **p < 0.01, ***p < 0.001

### 3.2 Mediating effects of impulsivity between breakfast skipping and mood symptoms

**Figure 1:**
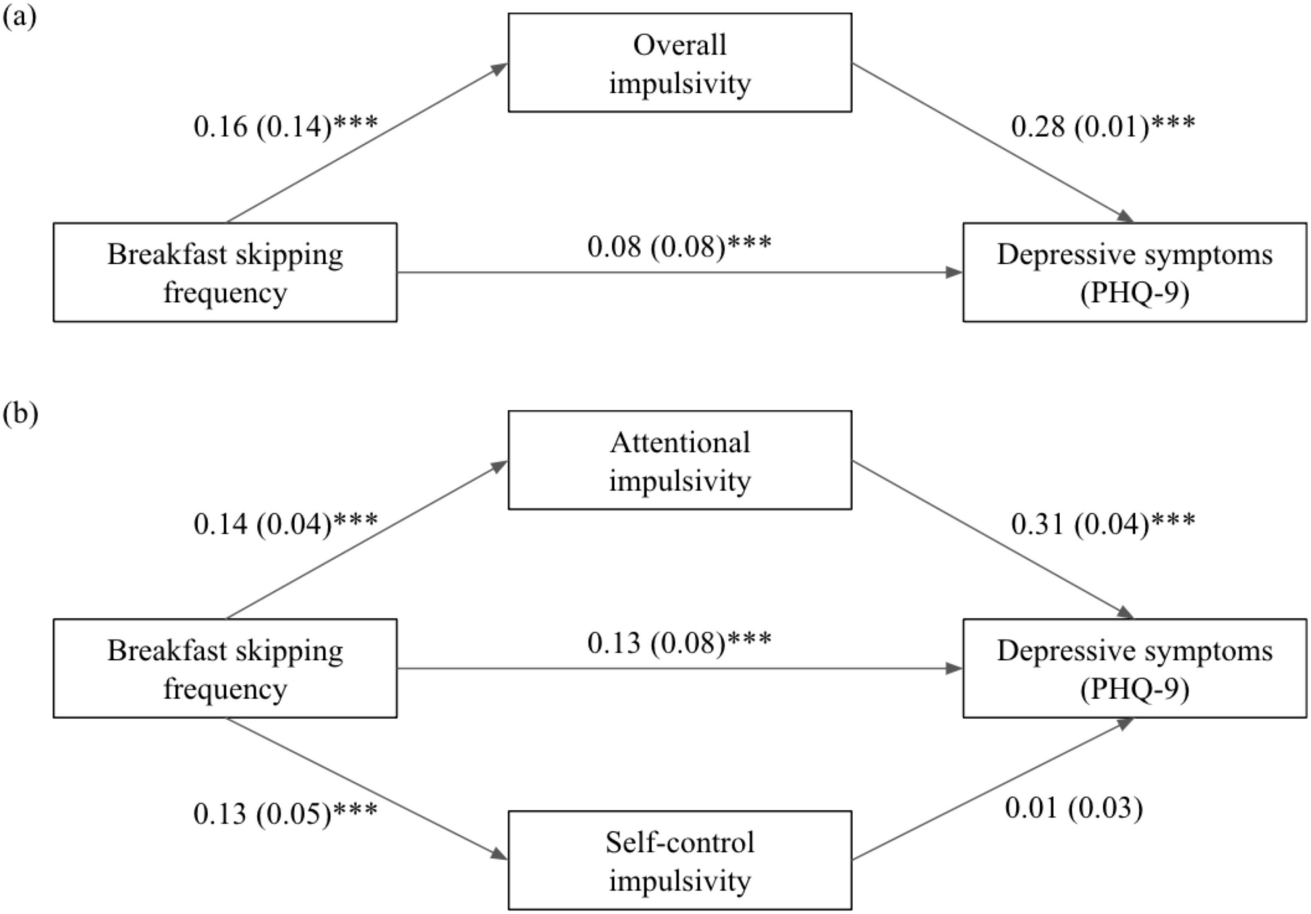
Diagram illustrating impulsivity as a putative mediator between breakfast skipping frequency and depressive symptoms, with **(a)** overall impulsivity, and specifically **(b)** attentional and self-control impulsivity, as the mediators. Sex, age, any psychiatric history, socioeconomic status, and symptoms of eating disorders were adjusted for in both models. Standardised coefficients are presented, with standard error in parentheses. PHQ-9 = Patient Health Questionnaire–9-item. ***p<0.001.

Two separate mediation models were constructed to examine the potential mediating effects of (i) overall impulsivity, as well as (ii) attentional and self-control impulsivity, between breakfast skipping frequency and depressive symptoms. With sociodemographic characteristics and eating disorder symptoms accounted for, overall impulsivity significantly mediated the effect of breakfast skipping on overall mood symptoms (*B* = 0.21, SE = 0.03, CI = 0.16–0.26) and accounted for 34.4% of the total effect in the model (**Figure 1a**). In the second model, a parallel mediation analysis was applied with both attentional and self-control impulsivity (**Figure 1b**). Results showed significant mediating effects of attentional impulsivity (*B* = 0.21, SE = 0.03, CI = 0.15–0.27) but not self-control impulsivity (*B* = 0.01, SE = 0.01, CI = -0.02–0.03) in the model. The two variables altogether explained 35.6% of the total effect, with 34.2% being explained by attentional impulsivity (**Figure 1b**).

Diagram illustrating impulsivity as a putative mediator between breakfast skipping frequency and depressive symptoms, with (a) overall impulsivity, and specifically (b) attentional and self-control impulsivity, as the mediators. Sex, age, any psychiatric history, socioeconomic status, and symptoms of eating disorders were adjusted for in both models. Standardised coefficients are presented, with standard error in parentheses. PHQ-9 = Patient Health Questionnaire–9-item. ***p<0.001

### 3.3 Sensitivity analysis

Additional analyses were conducted in the youth sample after excluding the subgroup of late risers (n = 2781). Findings from all analyses overall remained unchanged and detailed in the **Supplementary Material** (**Supplementary Tables S2**–**S4**, **Supplementary Figure S1**).

## 4 Discussion

This study was the first initiative to examine breakfast consumption habits in a household-based epidemiological youth sample and the possible role of various impulsivity domains between breakfast skipping and mental health symptoms. We found that around one-third of young people (33%) in Hong Kong consume breakfast daily, while nearly 15% completely skip breakfast on a regular basis. Confirming previous studies, we found more frequent breakfast-skipping patterns to be associated with elevated depressive symptoms. Aside from its relationship with overall impulsivity, we found breakfast skipping to be specifically associated with greater attentional and self-control impulsivity, among which attentional impulsivity significantly mediated the relationship between breakfast skipping and depressive symptomatology in young people. These observations have implications for future research and practice.

Despite often being referred to as one of the most important meals of the day, skipping breakfast has become a common lifestyle in modern society (5). While it may be a possible approach to reducing overall energy intake (43), studies have shown the associations of breakfast skipping with elevated risks of adverse health outcomes (6). The importance of regular breakfast consumption has thus been emphasised by governments and organisations, including the World Health Organization (44). While further controlled longitudinal studies are needed to establish the effects of breakfast on the course of depression (e.g., onset, recurrence, and relapse) and its mechanisms, our present study provided a perspective to suggest that attentional impulsivity, in particular, plays a role in the relationships between breakfast skipping and depressive symptoms.

Attentional impulsivity, as assessed using the BIS-11, is generally defined as “an inability to focus attention or concentrate” (45) and covers items such as “don’t pay attention” and “can concentrate easily” (reversed). Indeed, attention is known to be a core building block of cognitive-perceptual functions, with the ability to sustain and control attention playing a crucial role in the manifestation of depressive and anxiety disorders (46, 47). There is also growing evidence in support of attention training (including mindfulness-based cognitive training) in reducing overall depressive and anxiety symptoms and repetitive negative thinking (i.e., rumination and worries) (48–51) – a major transdiagnostic mechanism identified to underly the onset and maintenance of the two internalising disorders (52, 53). The need to give more consideration to the different domains of attention in depressive disorders and other mental health problems has also recently been highlighted (26). Our study contributed to this line of inquiry by showing not only its influences on depressive and anxiety symptoms but also the negative effects (at least to a small degree) of breakfast skipping on attentional control. Whether this relationship is explained by related physiological and metabolic mechanisms or other factors remains to be investigated in future work.

The present study has multiple strengths. The use of an epidemiological design ensured the representativeness of the findings in the youth population, which were generally aligned with previous studies. We also accounted for the influences of major confounding factors, such as demographics, socioeconomic status, psychiatric history, eating disorder symptoms, as well as rising time, which add confidence to the robustness of findings. Together with previous observations, the findings suggest that incorporating breakfast consumption as part of existing clinical interventions (e.g., as a form of behavioural activation) and lifestyle intervention for mental disorders alongside other approaches [e.g., sleep-based interventions (54), and nature experiences (55)] could have beneficial effects. Investing in more rigorous controlled trial studies to evaluate its implications is encouraged.

We note several limitations. First, the cross-sectional nature of the study could not provide definite information on the direction of causality among the variables. With appetite change being a common symptom of depression, it is possible that depressive symptoms could have played a role in the patterns of breakfast skipping we observed in the youth sample. While a number of longitudinal studies have shown a relationship between breakfast skipping and prospective depressive symptoms in young people and adults (8, 9), it would be helpful for future work to build on our study to elucidate the longitudinal, causal pathways among breakfast consumption, impulsivity (particularly attentional impulsivity and control), and mental health outcomes using a combination of experimental and longitudinal observation study designs. Further, while the BIS-11 is a widely adopted self-reported measure of impulsivity, incorporating laboratory-based tests as complementary assessments may offer additional information about specific patterns of associations between breakfast consumption/skipping and the cognitive and behavioural domains of impulsivity, respectively (56).

Contrary to findings from a previous systematic review (7), we did not find breakfast skipping to be associated with anxiety symptoms. Given that the effect sizes in our study were generally weak overall, whether breakfast consumption would be similarly or differentially related to depressive and anxiety symptom dimensions cannot be firmly concluded. The use of a retrospective measure for breakfast consumption can contribute to potential recall bias and may be a reason for the weak effect sizes observed. Nevertheless, the current findings may serve as a basis for future work to replicate and test. Aside from longitudinal studies and randomised controlled trials, the experience sampling method may be considered to pinpoint whether daily breakfast consumption patterns would contribute to subsequent attentional control and mood states, whilst accounting for contextual factors in the real-world setting (57, 58). Qualitative studies should also be conducted to unravel the reasons behind breakfast skipping among young people and, in turn, to inform more personalised intervention approaches and improve intervention uptake. Given the challenges in health behaviour change (59), aside from designing theory and empirically informed interventions, co-creating approaches with young people to facilitate the building of breakfast-eating habits is warranted.

Lastly, we acknowledge that specific components of breakfast [e.g., energy intake, nutrients, portion sizes (1)] could moderate the associations between consumption and health outcomes. A single-item measure of breakfast, similar to previous work (5, 11), can be beneficial for large-scale population screening and in time-limited settings. Nevertheless, it would be worthwhile for future studies to elucidate how each of these components affects physical and mental health and whether differential attention, neurobiological, physiological, and metabolic pathways would mediate their respective associations. These findings would be helpful in informing more effective interventions and providing policymakers with more specific health promotion strategies.

Mental health problems, particularly depression, are among the leading causes of the global burden of disease faced by young people and have considerable implications at individual, interpersonal, and socioeconomic levels. Although additional research is needed to elucidate the pathways and mechanisms underlying breakfast skipping, given problems of service non-utilisation among youths and insufficient manpower in psychiatric or psychological settings, encouraging regular breakfast consumption among young people may act as a simple, low-stigma, low-cost, and scalable strategy with physical, cognitive, and mental health benefits.

## Data Availability

The de-identified raw data supporting the conclusions of this article will be made available by the authors upon reasonable request, without undue reservation.
