# Supplementary material for: Breakfast skipping and depressive symptoms in an epidemiological youth sample in Hong Kong: the mediating role of reduced attentional control
Source: Front Psychiatry. 2025 May 22;16:1574119. doi: 10.3389/fpsyt.2025.1574119 (PMC12141854; doi:10.3389/fpsyt.2025.1574119)
Supplement: Supplementary file 1 [file DataSheet1.pdf]

Supplementary Material

Breakfast skipping and depressive symptoms in an epidemiological youth sample in Hong Kong: the mediating role of reduced attentional control

Table S1. Psychiatric conditions of young people with any psychiatric history in the sample.. 2
Table S2. Unweighted patterns of breakfast skipping in early and late rising young people... 4
Table S3. Sample characteristics after excluding the youth subsample with late rising time...4
Table S4. Correlations between breakfast skipping frequency, impulsivity, mental health symptoms, and functioning (after excluding the youth subsample with late rising time)..5
Figure S1. Attentional impulsivity as a mediator between breakfast skipping and depressive symptoms......................... 6

Table S1. Psychiatric conditions of young people with any psychiatric history in the sample (n = 273)

Psychiatric condition	n (%)
**Bipolar disorder**	9 (3.3%)
Comorbid with:	
Bipolar disorder, Borderline personality disorder	1 (0.4%)
Bipolar disorder, Major depression	1 (0.4%)
Bipolar disorder, Major depression, Generalised anxiety, Post-traumatic stress disorder	1 (0.4%)
Bipolar disorder, Social anxiety disorder	1 (0.4%)
**Eating disorders**	4 (1.5%)
Comorbid with:	
Eating disorders, Adjustment disorder	1 (0.4%)
Eating disorders, Generalised anxiety, Panic disorder	1 (0.4%)
**Generalised anxiety**	27 (9.9%)
Comorbid with:	
Generalised anxiety, Bipolar disorder	1 (0.4%)
Generalised anxiety, Major depression	1 (0.4%)
Generalised anxiety, Major depression, Obsessive-compulsive disorder	1 (0.4%)
Generalised anxiety, Panic attacks	1 (0.4%)
Generalised anxiety, Mild depression	1 (0.4%)
**Major depression**	71 (26%)
Comorbid with:	
Major depression, Generalised anxiety	25 (9.2%)
Major depression, Generalised anxiety, Bipolar disorder	1 (0.4%)
Major depression, Generalised anxiety, Borderline personality disorder	2 (0.7%)
Major depression, Generalised anxiety, Eating disorders	1 (0.4%)
Major depression, Generalised anxiety, Obsessive-compulsive disorder	1 (0.4%)
Major depression, Generalised anxiety, Panic disorder	1 (0.4%)
Major depression, Eating disorders	3 (1.1%)
Major depression, Obsessive-compulsive disorder	1 (0.4%)
Major depression, Obsessive-compulsive disorder, Persecutory paranoia	1 (0.4%)
Major depression, Social anxiety disorder	1 (0.4%)
Major depression, Post-traumatic stress disorder	1 (0.4%)
Major depression, Borderline personality disorder	1 (0.4%)
Major depression, Panic attacks	1 (0.4%)
**Neurodevelopmental disorders**	61 (22.3%)
Comorbid with:	
Neurodevelopmental disorders, Bipolar disorder	2 (0.7%)
Neurodevelopmental disorders, Borderline personality disorder	1 (0.4%)
Neurodevelopmental disorders, Conduct disorder	1 (0.4%)
Neurodevelopmental disorders, Generalised anxiety	2 (0.7%)
Neurodevelopmental disorders, Generalised anxiety, Social anxiety disorder	1 (0.4%)
Neurodevelopmental disorders, Major depression	4 (1.5%)
Neurodevelopmental disorders, Major depression, Borderline personality disorder	1 (0.4%)
Neurodevelopmental disorders, Major depression, Generalised anxiety, Borderline personality disorder	1 (0.4%)
Neurodevelopmental disorders, Obsessive-compulsive disorder	1 (0.4%)
**Psychosis**	3 (1.1%)
Comorbid with:	
Psychosis, Adjustment disorder	1 (0.4%)

Psychosis, Bipolar disorder	1 (0.4%)
Psychosis, Major depression	4 (1.5%)
Psychosis, Major depression, Generalised anxiety	1 (0.4%)
Psychosis, Major depression, Generalised anxiety, Obsessive-compulsive disorder	1 (0.4%)
**Others**	
**Anxiety disorder (not specified)**	1 (0.4%)
**Adjustment disorder**	4 (1.5%)
**Borderline personality disorder**	1 (0.4%)
**Conduct disorder**	1 (0.4%)
**Mild depression**	1 (0.4%)
**Mixed anxiety-depressive disorder**	4 (1.5%)
**Mood disorders**	1 (0.4%)
**Obsessive-compulsive disorder**	2 (0.7%)
**Panic attack/disorder**	2 (0.7%)
**Post-traumatic stress disorder**	1 (0.4%)
**Sleep disorder**	1 (0.4%)
**Social anxiety disorder**	2 (0.7%)
**Not specified**	4 (1.5%)

Table S2. Unweighted patterns of breakfast skipping in early and late rising young people

	Whole sample (n = 3154)	Early risers (n = 2781)	Late risers (n = 373)	*p*
Breakfast skipping patterns				<0.001
Eat breakfast daily	1022 (32.4%)	996 (35.8%)	26 (7.0%)	
Often eat breakfast	869 (27.6%)	817 (29.4%)	52 (13.9%)	
Rarely eat breakfast	780 (24.7%)	622 (22.4%)	158 (42.4%)	
Never eat breakfast	483 (15.3%)	346 (12.4%)	137 (36.7%)	

Note. Values are presented in the form of n (%).

As expected, young people with a later rising time (at 12 pm or after) were less likely to report eating breakfast on a daily basis (**Table S2**). Further analyses were thus conducted after excluding the subsample of late risers as a sensitivity analysis to confirm the associations observed.

Table S3. Sample characteristics after excluding the youth subsample with late rising time

	Subsample (n = 2781)	Breakfast consumption (daily/intermittent) (n = 2435)	Breakfast skipping (no consumption) (n = 346)	*p*
Sociodemographic variables and covariates				
Female sex, n (%)	1831 (58.1%)	1427 (58.6%)	211 (61%)	0.40
Age	19.8 (2.8)	**19.8 (2.8)**	**20.1 (2.6)**	**0.026**
Any psychiatric history, n (%)	273 (8.7%)	**200 (8.2%)**	**40 (11.6%)**	**0.038**
Any government subsidy received, n (%)	296 (9.4%)	228 (9.4%)	35 (10.1%)	0.66
Eating disorder symptoms (EDE-Q)	1.04 (1.00)	**1.00 (0.97)**	**1.17 (1.14)**	**0.003**
Impulsivity symptoms				
Overall impulsivity (BIS-11)	63.22 (8.49)	**62.92 (8.44)**	**65.34 (8.54)**	**<0.001**
Attention	10.89 (2.22)	**10.82 (2.22)**	**11.34 (2.18)**	**<0.001**
Cognitive instability	5.69 (1.60)	**5.64 (1.59)**	**6.05 (1.58)**	**<0.001**
Motor	13.61 (2.87)	**13.53 (2.82)**	**14.17 (3.13)**	**<0.001**
Perseverance	7.16 (1.52)	**7.13 (1.52)**	**7.32 (1.51)**	**0.036**
Self-control	13.98 (3.10)	**13.91 (3.10)**	**14.42 (3.02)**	**0.004**
Cognitive complexity	11.89 (2.21)	11.87 (2.19)	12.03 (2.36)	0.22
Mental health symptoms				
Depressive symptoms (PHQ-9)	6.43 (5.20)	**6.22 (5.09)**	**7.90 (5.71)**	**<0.001**
Anxiety symptoms (GAD-7)	4.74 (4.53)	**4.63 (4.48)**	**5.55 (4.82)**	**<0.001**
Functioning				
Days of reduced productivity	2.26 (4.77)	**2.12 (4.53)**	**3.20 (6.14)**	**0.002**
Days of lost productivity	0.38 (2.01)	**0.36 (1.93)**	**0.47 (2.47)**	0.35
Social and occupational functioning (SOFAS)	82.67 (7.71)	**82.94 (7.62)**	**80.82 (8.05)**	**<0.001**

Note. Values are presented in the form of mean (SD) or n (%). Statistics significant at the level of $p < 0.05$ are in boldface. BIS = Barratt Impulsiveness Scale; EDE-Q = Eating Disorder Examination Questionnaire; GAD-7 = Generalized Anxiety Disorder–7-item; PHQ-9 = Patient Health Questionnaire–9-item; SOFAS = Social and Occupational Functioning Assessment Scale.

Table S4. Correlations between breakfast skipping frequency, impulsivity, mental health symptoms, and functioning (after excluding the youth subsample with late rising time)

	1	2	3	4	5	6	7	8	9	10	11	12	13
Breakfast skipping													
1. Breakfast skipping frequency	–	–	–	–	–	–	–	–	–	–	–	–	–
Impulsivity													
2. Overall impulsivity (BIS-11)	**0.15***	–	–	–	–	–	–	–	–	–	–	–	–
3. Attention	**0.12***	**0.74***	–	–	–	–	–	–	–	–	–	–	–
4. Cognitive instability	**0.10***	**0.50***	**0.37***	–	–	–	–	–	–	–	–	–	–
5. Motor	**0.09***	**0.61***	**0.27***	**0.41***	–	–	–	–	–	–	–	–	–
6. Perseverance	0.03	**0.52***	**0.29***	**0.20***	**0.16***	–	–	–	–	–	–	–	–
7. Self-control	**0.11***	**0.73***	**0.54***	**0.11***	**0.17***	**0.30***	–	–	–	–	–	–	–
8. Cognitive complexity	**0.07***	**0.56***	**0.27***	0.00	**0.15***	**0.24***	**0.36***	–	–	–	–	–	–
Mental health symptoms													
9. Depressive symptoms (PHQ-9)	**0.14***	**0.34***	**0.37***	**0.36***	**0.11***	**0.20***	**0.20***	**0.11***	–	–	–	–	–
10. Anxiety symptoms (GAD-7)	**0.09***	**0.24***	**0.28***	**0.36***	**0.07***	**0.15***	**0.09***	**0.07***	**0.75***	–	–	–	–
Functioning													
11. Days of reduced productivity	**0.06****	**0.13***	**0.15***	**0.18***	0.02	**0.08***	**0.07***	0.03	**0.41***	**0.37***	–	–	–
12. Days of lost productivity	0.01	**0.10***	**0.09***	**0.15***	**0.04***	**0.06****	0.04	0.01	**0.25***	**0.24***	**0.35***	–	–
13. Social and occupational functioning (SOFAS)	**-0.08***	**-0.16***	**-0.15***	**-0.16***	-0.03	**-0.14***	**-0.11***	**-0.07***	**-0.39***	**-0.36***	**-0.30***	**-0.23***	–

Note. Findings are based on the youth sample after excluding late risers (n = 2781). Statistics significant at the level of $p < 0.05$ are in boldface. BIS = Barratt Impulsiveness Scale; GAD-7 = Generalized Anxiety Disorder–7-item; PHQ-9 = Patient Health Questionnaire–9-item; SOFAS = Social and Occupational Functioning Assessment Scale.

* $p < 0.05$, ** $p < 0.01$, *** $p < 0.001$

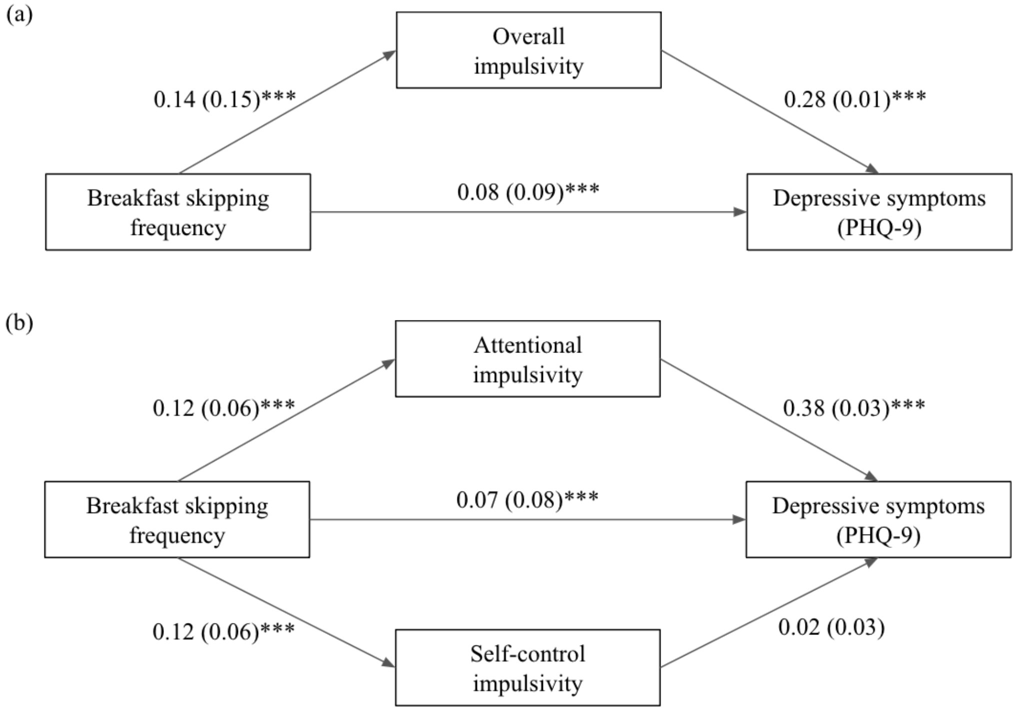

Figure S1. Attentional impulsivity as a mediator between breakfast skipping and depressive symptoms
Two mediation models were conducted in the youth sample after excluding late risers (n = 2781). Diagram illustrating impulsivity as a putative mediator between breakfast skipping frequency and depressive symptoms, with (a) overall impulsivity, and specifically (b) attentional and self-control impulsivity, as the mediators. Sex, age, any psychiatric history, socioeconomic status, and symptoms of eating disorders were adjusted for in both models. Standardised coefficients are presented, with standard error in parentheses. PHQ-9 = Patient Health Questionnaire–9-item. ***$p<0.001$

Two separate mediation models were constructed to examine the potential mediating role of (i) overall impulsivity, as well as (ii) attentional and self-control impulsivity, between breakfast skipping frequency and depressive symptoms, after excluding the subsample of late risers. With sociodemographic characteristics and eating disorder symptoms accounted for, overall impulsivity significantly mediated the effect of breakfast skipping on overall mood symptoms (B = 0.20, SE = 0.03, CI = 0.14–0.26) and accounted for 32.4% of the total effect in the model (**Fig S1a**). Parallel mediation analysis with both attentional and self-control impulsivity was then conducted (**Fig S1b**). Results showed that attentional impulsivity (B = 0.24, SE = 0.04, CI = 0.17–0.31), but not self-control impulsivity (B = 0.01, SE = 0.01, CI = -0.01–0.03), was a significant mediator in the model (**Fig 1b**). The two variables altogether explained 40.3% of the total effect in the model, with 38.6% being explained by attentional impulsivity. All patterns of associations were comparable to the full sample analysis as reported in the main text.
